# Conformational B-Cell Epitope Prediction Method Based on Antigen Preprocessing and Mimotopes Analysis

**DOI:** 10.1155/2015/257030

**Published:** 2015-01-29

**Authors:** Pingping Sun, Haixu Ju, Baowen Zhang, Yu Gu, Bo Liu, Yanxin Huang, Huijie Zhang, Yuxin Li

**Affiliations:** ^1^School of Computer Science and Information Technology, Northeast Normal University, Changchun 130117, China; ^2^National Engineering Laboratory for Druggable Gene and Protein Screening, Northeast Normal University, Changchun 130024, China; ^3^Key Laboratory of Intelligent Information Processing of Jilin Universities, Northeast Normal University, Changchun 130117, China; ^4^Faculty of Physical Education, Northeast Normal University, Changchun 130024, China

## Abstract

Identification of epitopes which invokes strong humoral responses is an essential issue in the field of immunology. Various computational methods that have been developed based on the antigen structures and the mimotopes these years narrow the search for experimental validation. These methods can be divided into two categories: antigen structure-based methods and mimotope-based methods. Though new methods of the two kinds have been proposed in these years, they cannot maintain a high degree of satisfaction in various circumstances. In this paper, we proposed a new conformational B-cell epitope prediction method based on antigen preprocessing and mimotopes analysis. The method classifies the antigen surface residues into “epitopes” and “nonepitopes” by six epitope propensity scales, removing the “nonepitopes” and using the preprocessed antigen for epitope prediction based on mimotope sequences. The proposed method gives out the mean *F* score of 0.42 on the testing dataset. When compared with other publicly available servers by using the testing dataset, the new method yields better performance. The results demonstrate the proposed method is competent for the conformational B-cell epitope prediction.

## 1. Introduction

In humoral immunization, a pathogenic antigen is recognized by an antibody or B-cell receptor (BCR) through some regions on the surface of the antigen that is commonly known as the B-cell epitope. Since humoral responses are induced by epitopes on the surface of antigen, rather than the whole antigen, it is important to locate these epitopes on antigen for the purpose of effective vaccine design. The most reliable methods for identification of an epitope are X-ray crystallography and NMR techniques, but they are time consuming and expensive. Candidate epitopes that are selected by computational methods prior to laboratory experiments can lead to both significantly reducing the experimental cost and substantially accelerating the identifying process [1].

A B-cell epitope can be categorized into two types by its spatial structure: liner epitope and conformational epitope. A liner epitope is composed of residues that are sequentially consecutive, whereas a conformational epitope consists of sequential segments that are brought together in spatial proximity when the corresponding antigen is folded. It has been reported that more than 90% of B-cell epitopes are discontinuous B-cell epitopes [2]; therefore, the prediction of conformational epitope is more significant.

Conformational epitope prediction methods can be divided into two categories: structure-based prediction and mimotope-based prediction. Structure-based prediction is through the 3D structure features of antigen and epitope propensity scales, such as geometric attributes and specific physicochemical properties. Mimotope-based prediction is a combinatorial method which requires both mimotope sequences and the 3D structure of antigen as input. These kinds of methods are essentially mapping mimotopes back to the surface of a source antigen to locate the best alignment sequences and predict possible epitopic regions.

In these years, many mimotope-based conformational B-cell prediction methods have been proposed, such as MEPS [3], 3DEX [4], MIMOX [5], Mapitope [6, 7], Sitelight [8], EpiSearch [9], PepSurf [10], Pep-3D-Search [11], and MimoPro [12]. These methods can be classified into two categories: sequence-sequence alignment methods and sequence-structure alignment methods [13]. Sequence-sequence alignment methods predict epitopes according the alignment of mimotope sequences and the antigen sequence, while sequence-structure alignment methods predict epitopes according to the alignment of mimotope sequences and the antigen structure. Sequence-structure alignment methods can be further divided into 4 kinds by the core idea of the alignment: motif-based methods, pairs-based methods, patch-based methods, and graph-based methods.

The latest mimotope-based conformational B-cell prediction method is MimoPro which was proposed by our team in 2011. MimoPro employs the idea of patch-based and graph-based searching. The core idea of MimoPro is a searching algorithm operated on a series of overlapping patches on the surface of antigen. These patches are then transformed to a number of graphs using an adaptable distance threshold (ADT) regulated by compactness factor (CF), a novel parameter proposed in the method. Then on each single patch, a complete search is conducted to guarantee the best alignment for each mimotope sequence. Dynamic programming and branch-bound methods are also adopted to both avoid repetition in searching and further narrow the search space.

Though the sensitivity of MimoPro is the highest so far, the specificity is not improved compared with other methods. In this paper, we present a new conformational B-cell epitope prediction method by antigen preprocessing and MimoPro searching. The method first absorbed the idea of both structure-based method and mimotope-based method. The performance of this method has been tested on 18 test cases which are relative large and complete datasets from the benchmark of MimoDB 2.0 [14]. The results showed the specificity of new method improved a lot. Moreover, it achieved the highest *F* score among all the available mimotope-based B-cell epitope prediction methods.

## 2. Materials and Method

### 2.1. Definitions

The definitions of an epitope inferred from the 3D structure of Ag-Ab complex are mainly based on either ASA or the contact area between residues of antigen and antibody. In the first state, an epitope is defined as the surface residue of antigen with ASA decreased more than a given threshold upon binding with the antibody, and 1 Å^2^ is frequently used. There are some tools to calculate ASA, and the usually used tool is NACCESS [15] or Surface Racer program [16]. In the second state, an epitope is defined as the residue of antigen which has a contact area above a given threshold upon interaction with the antibody, while the value 4 Å is frequently used. Among these two ways of definition the second one is generally accepted and applied. Ponomarenko tested in his paper that choosing which way to define epitope may not influence the results [17]. In this paper we define epitopes by the first way. In addition, we consider an amino acid residue as a surface residue if the RSA (relative accessible surface area) of its side chain is greater than 5% with 1.4 Å probe radius.

### 2.2. Datasets

The training datasets are from the representative Ag-Ab complexes in Ponomarenko and Bourne, the protein docking Benchmark 2.0 [18], and the testing datasets in the relevant papers [19–25]. We selected all Ag-Ab complexes and excluded the redundant structures and also excluded the ones which have more than one antigen chain. The 3D structure of the complexes is obtained from the PDB [26]. Finally, 150 Ag-Ab complexes with only one antigen chain were obtained as the training dataset. This dataset is used for machine learning in the step of antigen preprocessing. The training datasets can be obtained upon request.

The testing dataset is from the Mimobench of MimoDB. MimoDB is an information portal to biopanning results of random libraries [27]. It is the latest and largest database for mimotopes. In version 2.0, it offers a benchmark for mimotope-based site mapping. We compile the testing datasets as our previous work [28], and at last 18 cases which have only one mimotope set for one complex structure and the number of antigen amino acids which is larger than 67 from this Mimobench were selected. In 18 testing cases there are 13 antigen-antibody complexes and 5 protein-protein interactions. The testing dataset is listed in Table 1. We use the testing datasets for verifying the effectiveness of the new method and comparing the prediction performance of different methods.

### 2.3. Algorithm

**Figure 1:**
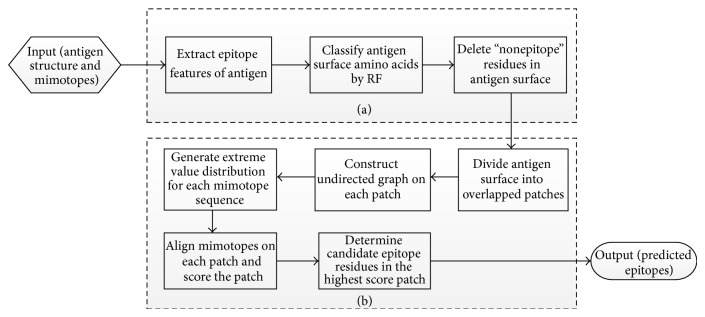
The algorithm flowchart of the method.

The algorithm flowchart of this method was shown in Figure 1. Input module is the start of a request submitted by user. Output module is the prediction results of the new method. The middle part is the flow of the method.

As shown in Figure 1, Figure 1(a) is the module of antigen processing, and Figure 1(b) is the module of mimotope-based epitope prediction which employs the core idea of our MimoPro.

#### 2.3.1. Antigen Processing

In 2011, we proposed MimoPro which is a novel mimotope-based conformational B-cell prediction method. Compared with other available mimotope-based methods, MimoPro achieved a better performance in sensitivity and precision; however, the specificity is lower than other methods. Since a good method should have a high score in both sensitivity and specificity, we analyzed the algorithm of MimoPro deeply and found that MimoPro predicted more candidate epitope residues, including true epitope residues and nonepitope residues. Hence, in this paper we developed MimoPro with the step of antigen preprocessing in which both reduce the number of predicted epitope residues which are not indeed the true ones and increasing the searching speed of MimoPro.

The implementation of antigen processing includes 3 steps: feature extraction, amino acids classification, and residues deletion.

Firstly, the method extracts 6 epitope related features as EPCES: residue epitope propensity, conservation score, side chain energy score, contact number, surface planarity score, and secondary structure composition. The detailed calculation of these six features is described in EPCES [29].

Secondly, the amino acids of antigen surface were classified into “epitope residue” and “nonepitope residues” according to the 6 features. The common machine learning methods can better handle the problems with nearly the same number of positive samples and negative samples. In fact, the real dataset in this study is imbalanced, and the instances from negative class take the majority of the data. There are commonly two approaches to deal with the imbalanced datasets: one is adding samples to minority class or assigning a high weight to the samples to minority class, and the other is downsizing the majority class. In the training dataset of this work, the rate of nonepitopes versus epitopes is about 8 : 1. To ensure the effectiveness of prediction, we deal with the imbalance data through the following ways.

1. Random sample from the negative data of the training dataset was executed to make the ratio of positive data and negative data 1 : 1 for *n* times; then there would be *n* new subsets of the training datasets.
2. Random forest model was built on each subset. Then for a new instance, *n* random forest models will give *n* results and the voting mechanism is utilized to make the final decision.

Random forest and data bootstrapping are implemented by Weka [30]. The purpose of antigen preprocessing is removing some real nonepitope residues to increase the prediction performance of the whole method. On the basis of this idea, we tested several combinations of parameters, and the parameters (*I* = 5, *K* = 0, *S* = 1) are adopted finally.

Lastly, “nonepitope” residues obtained in the above step were removed in the surface of antigen, and this preprocessed antigen would be taken as the input of epitope prediction module.

#### 2.3.2. Epitope Prediction

We use our prediction algorithm which is known as MimoPro for mimotopes analysis when the preprocessing of antigen finished. As shown in Figure 1, the core algorithm includes five steps: dividing antigen surface into overlapped patches, constructing undirected graph on each patch, generating extreme value distribution for each mimotope sequence, aligning mimotopes on each patch and scoring patch, and determining candidate epitope residues in the highest score patch.

1. As usually used, the number of amino acids in an antigen surface patch of given size is constant and may contain fixed number of amino acids in epitope and nonepitope. However, this may take an obvious defect. Different protein has different structures; even the same protein may have different domains. The space compactness of these regions has big diversity; hence an efficient patch should be “big” enough to contain the meaningful edges in sparse region and also be “small” enough to prune the false edges in compact region. MimoPro solves this problem; it generates overlapped patches with variable number of amino acids in it according to a compactness factor (CF). The presence of CF also makes next searching step simpler and faster.
2. For every antigen surface patch, we take each residue as a vertex and the useful connections which were determined in the above step were taken as edges to construct surface undirected graph.
3. Then the method needs to find the best matched path for each mimotope sequence in each surface patch graph. Since these paths may have different lengths, to assess the similarity between a path and a mimotope sequence and to give consensus scores to these paths with different lengths, MimoPro employs a statistical scoring norm called *P* value which is derived from the extreme value distribution, and the parameters are fitted from the empirical distribution [31].
4. Dynamic programming and branch and bound method were employed in the step of aligning mimotopes on each patch. Dynamic programming method ensures the searching is complete; and the branch and bound method ensures the searching is efficient. Then after this step, every patch was scored according to the matching paths. Then the residues in the highest score patch are retained as the candidate epitopes.

The detail of the algorithm was described in our previous work [12]. Then the output of this module was taken as the prediction results of the whole method.

#### 2.3.3. Performance Measures

The performance of the prediction methods is scored by the commonly used measures: sensitivity (Sen), specificity (Spe), precision (PPV), Matthews correlation coefficient (MCC), accuracy (ACC), and *F*-measure. The measures are computed as follows:

(1) Sen (sensitivity  or  true  positive  rate)=TPTP+FN,Spe specificity=TNFP+TN,PPV positive  predictive  value  or  precision=TPTP+FP,MCC (Matthews  correlation  coefficient) =TP×TN−FP×FNTP+FPTP+TNTN+FPTN+FN,ACC accuracy=TP+TNTP+TN+FP+FN,F (F-measure)=2×PPV×SenPPV+Sen,

where TP is the number of predicted epitope residues proven to be the true epitope residues. FP is the number of predicted epitope residues proven not to be the true epitope residues. TN is the predicted nonepitope residues proven not to be the true epitope residues. FN is the number of predicted nonepitope residues proven to be the true epitope residues. In this paper, we took the number of antigen surface amino acids as TP + FP + FN + TN for calculating the above performance measures.

## 3. Results and Discussion

### 3.1. Performance of the Method Based on Antigen Processing

The results on the testing dataset of this method are shown in Table 2. Sensitivity, specificity, PPV, MCC, ACC, and *F* scores were listed. To give a comparison with MimoPro, we also tested MimoPro and listed the performance measures together with the new method.

As seen from Table 2, the number of predicted epitope of this method was less than MimoPro for most cases, and it leads to the improvement of the specificity of the new method. The average specificity score on testing dataset has reached 81% which is higher than MimoPro. The average sensitivity score of this method decreased slightly, and the reason is that there is no validated feature or feature combination that can distinguish epitope residues from nonepitope residues completely so far. That is, the removed residues which we took as “nonepitope” residues in the step of antigen preprocessing may contain more or less true epitope residues. Taking vascular endothelial growth factor (PDB id: 1BJ1) as an example, the native epitope includes two consecutive segments (I80 M81 R82 I83 K84, and H86 Q87 G88 Q89 H90 I91 G92 E93 M94) and five isolated amino acids (F17 Y21 Y45 K48 Q79). In the step of antigen preprocessing, 15 residues were removed (20V 27H 28P 40P 42E 47F 56R 58G 67E 68C 71T 78M 82R 98Q 100N), including R82, which is the true epitope residue. While predicted only by MimoPro, 32 candidate epitope residues (V33 D34 F36 Q37 E38 Y39 P40 D41 E42 I43 E44 Y45 I46 F47 K48 P49 S50 C51 M78 M81 R82 I83 K84 P85 Q87 G88 Q89 H90 G92 E93 M94 S95) were predicted which covers the residue R82. The new method predicted one less true epitope residue than MimoPro. Hence, the sensitivity of the new method is slightly lower than MimoPro.

However, sensitivity and specificity are not complete for evaluating the performance of one method. The PPV have more predictive validity. As seen from Table 2, the average PPV values of this method were higher than that of MimoPro, which not only illustrated that the overall performance of this method is superior to MimoPro but also indicated that the improvement of this study is effective.

**Figure 2:**
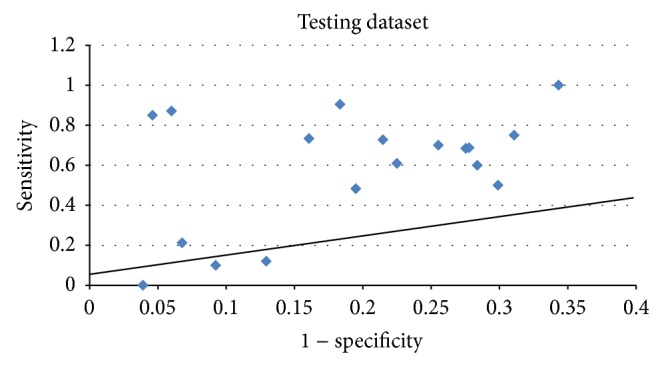
Sensitivity versus 1− specificity scores of the method on testing dataset.

Further, we draw Figure 2 to give directly relations between sensitivity and 1− specificity of this method. From Figure 2, we can see that for most test cases this method can precisely localize epitope regions. The predicted results of the method are totally better than random prediction. For 3BT1 and 2HYM, the two points on the *x*-axis, the method displayed no predictive ability. The main reason is that the second step of the method predicts no epitope residues according to mapping the mimotopes to the surface of the antigen.

### 3.2. Performance of This Method Based on RF and SVM

We use RF from Weka for classifying antigen surface residues in this work. To answer whether different machine learning method would influence the prediction performance of the method, we also employed SVM for classifying the surface amino acids of antigen, and latest version of LibSVM [30] was exploited in this work. For both methods, we tried lots of combinations of parameters. Several better results of the two methods are listed in Table 3.

For RF, we tested lots of combinations of parameters. Table 3 listed three results with top three PPV scores. For SVM, we deal with this imbalance through three ways: partition of the training data into blocks as introduced by Fu et al. [32], setting a weight value for positive instances, and both. In addition, binary-class cross validation with AUC was used. Table 3 gave the best results for each solution. As seen from Table 3, no matter if we use RF or SVM for antigen preprocessing, the performance of the new method performed better than MimoPro whose results have been listed in Table 2. Moreover, we can see that the predicted ability of RF on the testing dataset was better than SVM, and this is the reason why we chose RF in the module of antigen preprocessing. Then compared with the different parameters combination of RF, we chose *I* = 5, *K* = 0, and *S* = 1 which gave the highest values of PPV for RF in the antigen preprocessing.

### 3.3. Comparison with Other Methods

**Figure 3:**
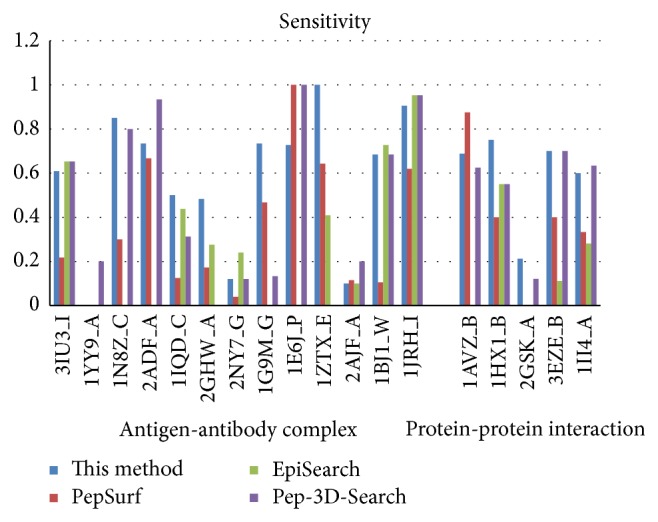
The sensitivity of each method on the testing dataset.

**Figure 4:**
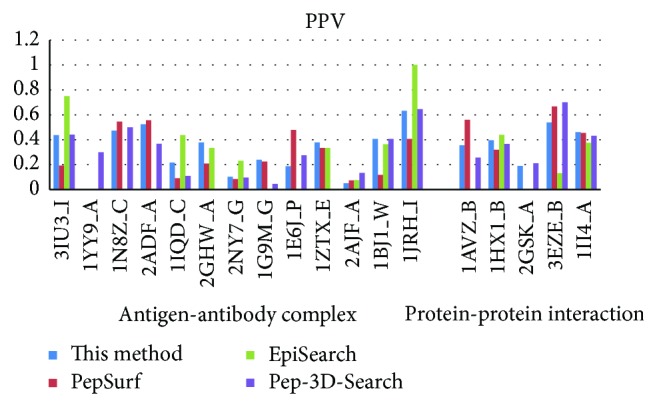
The PPV of each method on the testing dataset.

**Figure 5:**
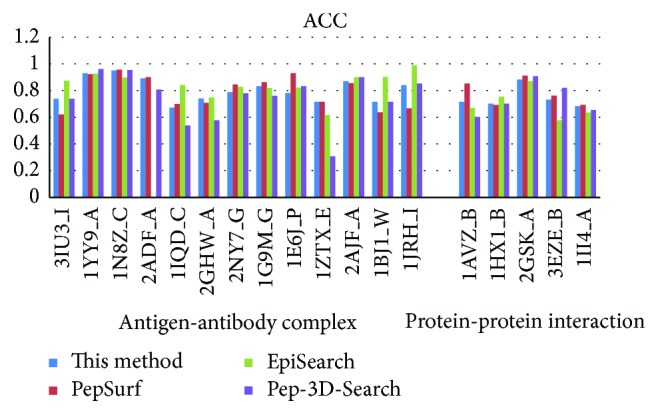
The ACC of each method on the testing dataset.

**Figure 6:**
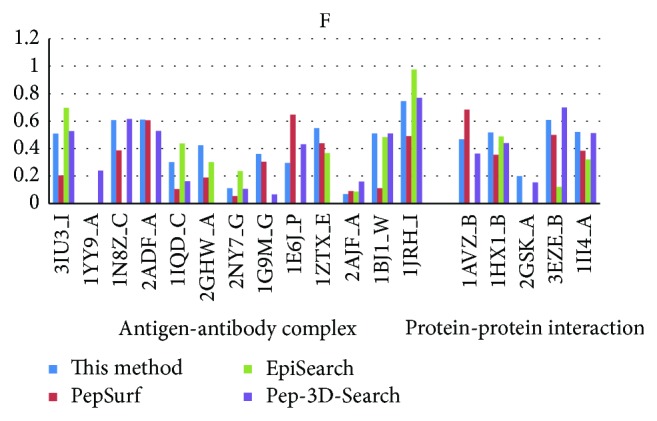
The *F* of each method on the testing dataset.

In recent years, there are several mimotope-based methods that have been proposed to predict conformational B-cell epitopes. In this work, we compared the new method with three other available mimotope-based conformational B-cell epitope prediction methods: PepSurf, EpiSearch, and Pep-3D-Search. These methods were tested in April of 2014, and the default parameters were adopted for each method. Figures 3, 4, 5, and 6 give the sensitivity, PPV, ACC, and *F* of each method on the testing dataset. 1ZTX, 1JRH, and 1WLP have no prediction results by EpiSearch due to the restriction of the method that the number of mimotope sequences cannot be larger than 30.

Further, we calculate the average values of these performance measures using the testing dataset for each method, respectively.  Table 4 gives the overall performance for each method. As shown in Table 4, the sensitivity of our method achieves 0.44 which is the same as the Pep-3D-Search and the highest among the 4 methods. The specificity is slightly lower than the EpiSearch which has the best specificity measure of 0.83 on this testing dataset. However, the new method was rated the best with a PPV of 0.33 which is improved a lot than the other methods.

In general, our new method demonstrates overall higher prediction accuracy than MimoPro and other three mimotope-based conformational B-cell epitope prediction methods on the testing dataset.

## 4. Conclusions

B-cell epitope prediction is important for vaccine design, development of diagnostic reagents, and interpretation of the antigen-antibody interactions on a molecular level. Localizing epitopes by experimental methods is expensive in terms of time, cost, and effort; therefore, computational methods feature for its low cost and high speed was employed to predict B-cell epitopes. In these years, lots of computational methods have been proposed for epitope prediction. These methods predict epitopes either by antigen structure or by mapping mimotopes to the original antigen surface. In this study, we proposed a new epitope prediction method based on antigen preprocessing by six epitope propensity scales and MimoPro searching. The performance of the method is superior to random prediction. Besides specificity, PPV measure improved a lot compared to MimoPro on the testing datasets. Compared with Pep-3D-Search, EpiSearch, and PepSurf, three other mimotope-based tools, testing results from the new method have shown that in most cases, it performed equal to or better than the what three methods did. On average from 18 test cases, performance of the new method indicated by sensitivity, PPV, and *F* value is better than that of Pep-3D-Search, EpiSearch, and PepSurf in epitope prediction. This implies that the new method is a viable alternative to, if not the preferred choice, all of PepSurf, Pep-3D-Search, EpiSearch, and MimoPro for epitope prediction in the same kind.

However, the new method is the first attempt to combine the idea of structure-based method and mimotope-based method, and the method is an improvement of our MimoPro. As seen from the results that for extremely difficult cases where amino acids forming the epitope include both consecutive segments and isolated amino acids, such as 3BT1 and 2HYM, the method failed in producing any useful mappings. This indicated where our method should be further improved although outcomes from PepSurf, EpiSearch, and Pep-3D-Search for the two test cases were not good either. Potentially this could be achieved through the following aspects in future. Firstly, selecting effective features or feature combination may potentially improve the performance of antigen processing. Secondly, to improve the performance of mapping mimotopes to the antigen surface, a more appropriate substitution matrix according to a specific application should be adopted so that graph rating is more meaningful to such application. In addition, intelligent searching algorithm could be modified so that the highly rated patches are searched first to make searching more efficient.

## Figures and Tables

**Table 1:** The detailed information of the testing dataset.

PDB_ID	Template chain	Target	Mimotope size	Reference
Antigen-antibody complex				
3IU3	I	Basiliximab	6 ∗ 9	17440057
1YY9	A	Cetuximab	4 ∗ 12	16288119
1N8Z	C	Herceptin	5 ∗ 12	15210798
2ADF	A	82D6A3, IgG	3 ∗ 8	12855711
1IQD	C	Anti-coagulation factor VIII monoclonal antibody BO2C11	27 ∗ 12	12676786
2GHW	A	80R	18 ∗ 15	16630634
2NY7	G	B12	17 ∗ 14, 1 ∗ 10, 1 ∗ 13	16940148
1G9M	G	Anti-gp120 monoclonal antibody 17b	1 ∗ 10, 10 ∗ 12	14596802
1E6J	P	13b5	14 ∗ 14, 2 ∗ 7	14596802
1ZTX	E	E16	3 ∗ 13, 19 ∗ 14	18760481
2AJF	A	SARS-coronavirus spike protein	18 ∗ 15	1116480
1BJ1	W	rhuMAb	36 ∗ 6, 3 ∗ 5, 2 ∗ 4	10543973
1JRH	I	A6, IgG1	59 ∗ 5	11123892

Protein-protein				
1AVZ	B	Fyn	8 ∗ 10,10 ∗ 12	7988556
1HX1	B	Heat shock cognate 71 kDa protein	8 ∗ 15	7649995
2GSK	A	Protein TONB	6 ∗ 9	16414071
3EZE	B	Protein (phosphotransferase system, HPR)	11 ∗ 6	10048929
1II4	A	Fibroblast growth factor receptor 2	30 ∗ 7	12032665

**Table 2:** The prediction results on testing datasets.

PDB_ID	MimoPro/this method								
	True epitopes	Predicted epitope	Sen	Spe	PPV	MCC	(Sen + Spe)/2	ACC	*F*
Antigen-antibody interactions									
3IU3_I	23	34/32	0.61/0.61	0.75/0.78	0.41/0.44	0.18/0.19	0.68/0.69	0.72/0.74	0.49/0.51
1YY9_A	15	43/18	0.00/0.00	0.91/0.96	0.00/0.00	−0.01/−0.01	0.45/0.48	0.88/0.93	0.00/0.00
1N8Z_C	20	38/36	0.90/0.85	0.95/0.95	0.47/0.47	0.14/0.13	0.93/0.90	0.95/0.95	0.62/0.61
2ADF_A	15	24/20	0.87/0.87	0.90/0.94	0.54/0.65	0.23/0.25	0.89/0.90	0.90/0.93	0.67/0.74
1IQD_C	16	40/37	0.56/0.50	0.68/0.70	0.23/0.22	0.08/0.07	0.62/0/60	0.66/0.67	0.32/0.30
2GHW_A	29	38/37	0.48/0.48	0.80/0.81	0.37/0.38	0.13/0.14	0.64/0.64	0.73/0.74	0.42/0.42
2NY7_G	25	30/29	0.12/0.12	0.87/0.87	0.10/0.10	0.00/0.00	0.49/0.50	0.78/0.79	0.11/0.11
1G9M_G	15	47/46	0.73/0.73	0.83/0.84	0.23/0.24	0.10/0.10	0.78/0.79	0.83/0.83	0.35/0.36
1E6J_P	11	45/43	0.73/0.73	0.77/0.79	0.18/0.19	0.08/0.08	0.75/0.76	0.77/0.78	0.29/0.30
1ZTX_E	14	39/37	0.10/0.10	0.63/0.66	0.36/0.38	0.24/0.24	0.81/0.83	0.69/0.72	0.53/0.55
2AJF_A	20	43/39	0.10/0.10	0.90/0.91	0.05/0.05	0.00/0.00	0.50/0.50	0.86/0.87	0.06/0.07
1BJ1_W	19	32/32	0.68/0.68	0.72/0.72	0.41/0.41	0.19/0.19	0.70/0.70	0.72/0.72	0.51/0.51
1JRH_I	21	31/30	0.95/0.90	0.82/0.82	0.65/0.63	0.38/0.36	0.88/0.86	0.85/0.84	0.77/0.75

Protein-protein interactions									
1AVZ_B	16	32/31	0.69/0.69	0.71/0.72	0.34/0.35	0.16/0.17	0.70/0.70	0.70/0.72	0.46/0.47
1HX1_B	20	38/38	0.75/0.75	0.69/0.69	0.39/0.39	0.20/0.20	0.72/0.72	0.70/0.70	0.52/0.52
2GSK_A	33	40/37	0.21/0.21	0.93/0.93	0.18/0.19	0.04/0.04	0.57/0.57	0.88/0.88	0.19/0.20
3EZE_B	20	27/26	0.75/0.70	0.74/0.74	0.56/0.54	0.29/0.27	0.75/0.72	0.75/0.73	0.64/0.61
1II4_A	30	41/39	0.60/0.60	0.69/0.72	0.44/0.46	0.18/0.19	0.64/0.66	0.66/0.68	0.51/0.52

Average			0.60/0.58	0.79/0.81	0.33/0.34	0.14/0.15	0.70/0.70	0.78/0.79	0.41/0.43

**Table 3:** The prediction performance of SVM and RF with different parameters.

Different parameters	Sen	Spe	PPV	MCC	(Sen + Spe)/2	ACC	*F*
RF							
*I* = 5, *K* = 0, *S* = 1	0.58	0.81	0.34	0.15	0.70	0.79	0.43
*I* = 10, *K* = 0, *S* = 1	0.56	0.83	0.34	0.14	0.69	0.80	0.42
*I* = 15, *K* = 0, *S* = 1	0.53	0.83	0.34	0.14	0.68	0.80	0.40
LibSVM							
Blocked	0.58	0.80	0.32	0.14	0.69	0.79	0.42
Weight	0.57	0.81	0.33	0.14	0.69	0.79	0.41
Blocked and weight	0.58	0.81	0.33	0.14	0.69	0.79	0.42

**Table 4:** The overall performance of the compared methods on testing dataset.

Methods	Sen	Spe	PPV	MCC	(Sen + Spe)/2	ACC	*F*
Pep-3D-Search	0.48	0.78	0.29	0.08	0.63	0.75	0.35
EpiSearch	0.31	0.89	0.28	0.09	0.60	0.70	0.19
PepSurf	0.36	0.86	0.26	0.07	0.61	0.79	0.31
This method	0.58	0.81	0.33	0.14	0.69	0.79	0.42
